# Ten Quick Tips for Using the Gene Ontology

**DOI:** 10.1371/journal.pcbi.1003343

**Published:** 2013-11-14

**Authors:** Judith A. Blake

**Affiliations:** Computational Biology and Bioinformatics, The Jackson Laboratory, Bar Harbor, Maine, United States of America; Whitehead Institute, United States of America

The Gene Ontology (GO) provides core biological knowledge representation for modern biologists, whether computationally or experimentally based. GO resources include biomedical ontologies that cover molecular domains of all life forms as well as extensive compilations of gene product annotations to these ontologies that provide largely species-neutral, comprehensive statements about what gene products do. Although extensively used in data analysis workflows, and widely incorporated into numerous data analysis platforms and applications, the general user of GO resources often misses fundamental distinctions about GO structures, GO annotations, and what can and can not be extrapolated from GO resources. Here are ten quick tips for using the Gene Ontology.

## Tip 1: Know the Source of the GO Annotations You Use

The GO site has the most comprehensive and current sets of annotations (http://www.geneontology.org/GO.downloads.annotations.shtml). All annotations are supported with evidence and source information. Hundreds of GO annotations are added daily to the GO database by over 15 major contributing groups. Annotations are also contained within external tools and applications, although these may not be updated often. If you are relying on external tools to provide GO resources for your data analysis, then it behooves you to know how comprehensive and current the GO annotations and ontology representations are that you are using. At the GO site, the most recent submission dates are listed, and most files are updated weekly. None of the files at the GO site are older than one year from last submission.

## Tip 2: Understand the Scope of GO Annotations

The GO annotation stream focuses on the capture of the knowledge about the functional activities of specific proteins, the larger biological role or process (such as photosynthesis) as part of which these specific functions collectively act, and the cellular locality where all this occurs. The terms defined for these components are represented by the “Molecular Function,” “Biological Process,” and “Cellular Component” domains of the overall Gene Ontology. Protein annotation associations with these terms capture our best knowledge about protein actions, and annotations capture different granularity of knowledge based on the experimental data available. All annotations are of value in understanding the scope of knowledge about a protein. Recent contextual extensions of annotations provide spatial and temporal aspects of these activities, although contextual details are not universally presented in web resources. GO editors are working together with developers of orthogonal ontologies such as the Cell Type Ontology, Life Stage Ontologies, CHEBI (chemical), and Anatomical Ontologies to provide improved representations of these contextual details. [1]–[3].

## Tip 3: Consider Differences in Evidence Codes

There are 21 evidence codes currently in use to document how data are obtained, and GO may adopt an evidence code classification in the near future. Some evidence codes indicate different classes of experiments such as “inferred by direct assay.” Some indicate different approaches to prediction from comparative analysis such as “inferred from sequence orthology.” Annotations generated by inferring (via orthology, ISO, for example) from one species to a second one are not used to infer back to the first species; this would be circular reasoning. Details about codes and their usage can be found here: http://www.geneontology.org/GO.evidence.shtml.

## Tip 4: Probe Completeness of GO Annotations

While the GO annotation corpus generally has excellent broad coverage of available knowledge about gene products via structurally based annotations from sources such as InterPro, the completeness of annotations derived from biomedical literature is uneven because there are not enough GO curators to keep them current on all fronts. The GO leadership and community requests determine priorities for comprehensive curation of biomedical literature. Requests from users for GO curatorial attention to particular sets of genes can generally be accommodated. Bottom line: the lack of a GO annotation does not mean that a gene product does not perform a particular function or act in a particular role; embrace this “open world” assumption [4].

## Tip 5: Understand the Complexity of the GO Structure

The GO structure, relations, and terminology are modified every day by GO ontology editors. New relations between terms are added as the GO refines the representation of biological knowledge. The addition of relationships such as “negative regulation of” provides distinct networks. Use of transitive closure to gather up all annotations relevant to your protein of interest gives the fullest picture of knowledge about your protein of interest and makes best use of the ontological structure. In some applications, subgraphs of GO may be more appropriate. GO editors work with other biomedical ontology developers to align and cross-reference these ontologies, and to improve knowledge representations. Learn more at http://www.geneontology.org/GO.contents.doc.shtml#ontology.

## Tip 6: Choose Analysis Tools Carefully

Hundreds of GO-focused applications and tools are available and are currently hosted on the NeuroLex site: http://neurolex.org/wiki/Category:Resource:Gene_Ontology_Tools. 

Term enrichment analysis, a common use of GO resources, is incorporated into many different applications and analysis tool sites. Tools are designed for different purposes such as “GO-slims” for grouping annotation sets by category and “Enrichment Tools” for statistical evaluation of gene sets. Different implementations/algorithms may give different results. Be especially vigilant to investigate how recent the GO files are that are embedding in data analysis tools. Several studies have evaluated the common algorithmic approaches and details to aid researchers in making intelligent tool-usage decisions [5], [6].

## Tip 7: Provide the Version of the Data/Tools Used

As mentioned in Tip 1, many tools incorporate GO annotations in their workflows, and of these, some do not provide versioning information of the tool or the annotation sets provided. Reporting the version of the data and tools used in computational analysis is essential in support of scientific reproducibility, a core paradigm of the scientific enterprise. File headers might provide information such as…

!gaf-version: 2.0

!software version:$Revision: 1.76 $

!date: 08/21/2012 $

## Tip 8: Seek Input from the GOC Community and Make Use of GOC Resources

The GO Consortium (GOC) is a large and active group bringing together a global functional genomics community. We are responsive to user requests for updates and explanations. We quickly respond to requests sent to http://www.geneontology.org/GO.contacts.shtml.

The GOC provides extensive documentation about ontology and annotation streams. Download the GO database, obtain ontology and annotation datasets in a variety of formats, join GOC social media sites or mailing lists, and review FAQ pages from www.geneontology.org. All GO resources are freely and openly available.

## Tip 9: Contribute to the GO

Find a questionable annotation or ontology node? Contact the GOC help desk and let us know (URL provided in Tip 8). Missing a term? Submit a SourceForge request at http://geneontology.sourceforge.net. Bring your expertise to update sections of the GO. You can also download GO tools and learn to construct and contribute annotations. Thanks for your help!

## Tip 10: Acknowledge the Work of the GO Consortium

The GOC depends on all the usual metrics to justify continued funding. As with any resource or data contributing to your success, remember to reference the GO resources in your publications. The 2000 *Nature Genetics* paper [7] is the core GO paper when citing the GO. You can additionally cite NAR database issue papers [1], [8].

## Conclusion

The Gene Ontology is a dynamic ontology-based resource that provides computationally tractable and human-digestible information about molecular systems. As one of the first and primary biomedical ontologies, the development of the GO pioneered the use of ontologies in computational biology. Intelligent use of GO resources ensures best results in advancing biological research.
